# Policy entrepreneurship in the reform of pediatric dentistry

**DOI:** 10.1186/s13584-017-0160-5

**Published:** 2017-06-30

**Authors:** Burton L. Edelstein, William R. Maas

**Affiliations:** 1 Dental Medicine and Health Policy and Management, Columbia University Medical Center, New York, NY USA; 2 Public Health Consultant, 11302 Old Club Road, North Bethesda, MD 20852 USA

**Keywords:** Dental care for children, Access to care, Financing dental care, Health policy, Policy entrepreneur, Philanthropy, Dental therapist, Dental care delivery

## Abstract

In a recently published IJHPR article, Cohen and Horev ask whether an individual who holds rightful governmental power is able to effectively “challenge the equilibrium” in ways that might “clash with the goals” of an influential group”. This question is raised within the context of a shift in governmental policy that imposed the potential for cost management by HMOs acting as financial intermediaries for pediatric dental care in an effort to provide Israeli children better access to affordable dental care. The influential group referred to consists of Israel’s private dentists and the individual seeking to challenge the equilibrium was an Israeli Minister of Health whom the authors consider to be a policy entrepreneur.

The Israeli health care system is similar to that of the United States in that private benefit plans and self-pay financing dominate in dental care. This is in contrast to the substantial role of government in the financing and regulation of medical care in both countries (with Israel having universal coverage financed by government and the US having government financing the care of the elderly and the poor as well as providing subsidies through the tax system for the care of most other Americans).

Efforts to expand governmental involvement in dental care in both countries have either been opposed by organized dentistry or have suffered from ineffective advocacy for increased public investment in dental care.

In the U.S., philanthropic foundations have acted as or have supported health policy entrepreneurs. The recent movement to introduce the dental therapist, a type of allied dental professional trained to provide a narrow set of commonly-needed procedures, to the U.S. is discussed as an example of a successful challenge to the equilibrium by groups supported by these foundations. This is a somewhat different, and complementary, model of policy entrepreneurship from the individual policy entrepreneur highlighted in the Cohen-Horev paper.

The political traction gained to change the equilibrium favored by organized dentistry – in both Israel and the U.S. - may reflect aspirations for care that is more accessible, patient-centered, accountable and equitable. Evolving aspirations may lead to policy changes to systematize the disparate, disaggregated dental care delivery system in both counties. A change in payment incentives to provide more value is being explored for medical care, and its expansion to dental care can be anticipated to be among the policies considered in the future.

## Background

In a recently published Israel Journal of Health Policy Research article, Cohen and Horev ask whether an individual who holds rightful governmental power is able to “challenge the equilibrium” in ways that might “clash with the goals of an influential group.” [1]. This question is raised within the context of a shift in governmental policy that imposed the potential for cost management by HMOs acting as financial intermediaries for pediatric dental care. The influential group referred to consists of Israel’s private dentists and the individual seeking to challenge the equilibrium was an Israeli Minister of Health whom the authors consider to be a policy entrepreneur.

This shift in policy, using HMOs to assure the provision of preventive services to all children in Israel, may seem modest to some. It did not seek to change the delivery system from private to public (i.e. governmental). It did not call on government to directly deliver care to under-served children. However, it was significant in that it addressed financial barriers to care for a large, vulnerable population and may also have set the wheels in motion to introduce accountability and value-based purchasing in Israeli pediatric dental care.

Cohen and Horev ask about the capacity of an individual to challenge the equilibrium; a related and no less important question is whether and when it is appropriate to do so. This question is multilayered and cannot be answered without consideration of political philosophy. Part of the answer may lie in the “Harm Principle” advanced by nineteenth century political philosopher John Stuart Mill [2], as part of his utilitarian philosophy. The principle posits that “the only purpose for which power [such as that held by the government-affiliated health policy entrepreneur] can be rightfully exercised over any member of a civilized community [i.e. the Israeli dentist], against his will, is to prevent harm to others.” This raises two questions. Exactly who is the “other,” the dentist or Israeli society? And does harm result from the policy entrepreneur’s action?

If the “other” is the private practice Israeli dentist Mill would reject the policy action and find this exercise of power to be wrongful. The private practice Israeli dentist, like his or her American counterpart, seeks preservation of a perceived professional prerogative of autonomy and control, feels threatened by imposition of a more government-regulated payment policy, and is concerned about potential income reduction, But if the “other” is Israeli children, Mill would approve the action as “rightful.” This population could benefit from the entrepreneur-induced payment policy through greater access to affordable dental care, and hence no harm is done (quite the opposite!).

It may be too early to determine the outcome of the policy change. However, if oral health of children is a primary good, a valued end in itself, then the means by which it is achieved in Israel or the U.S., relative to professional preferences, is secondary, unless dentists respond by not participating in public insurance. Inherent in this controversy is the very concept of professionalism – a challenging concept that calls upon dentists as professionals to put the health interest of others ahead of their own material interests, even in the face of practice demands and expectations.

## Political philosophy, equilibrium and the policy entrepreneur

Deciding on which consideration of the “other” is germane in the specific case of changing Israeli dental payment policy requires context, specifically the context of the Israeli approach to health policy. Countries vary significantly in the approach they take in determining their healthcare delivery and payment systems. Which political philosophic approach they choose dictates a cascade of decisions that ultimately characterize the systems they create. Examining international variation in pediatric oral health policy, Lowell-Shlansky and colleagues have proposed a conceptual model that associates political philosophy with financing approaches, financing sources, payment mechanisms, and delivery systems to explain pediatric oral health services [3]. Some countries, like Germany and Denmark, align these components directly. Germany takes a conservative approach that is matched with a Bismarkian financing system, relying mostly on private payment, and a mostly independent private delivery system while providing coverage for pediatric dental care. Denmark, similarly assures dental care for children, but does so through an approach that is socialistic, based on a Nordic financing system funded and paid publicly and delivered primarily through government clinics. Both countries approach medical and dental care through the same mechanisms.

What is so curious about the Israeli healthcare system is that it – like the U.S. system – bifurcates medical and dental care in ways that leave it internally conflicted or at least inconsistent. In both countries, governmental insurance, as a significant source of funding that influences even private insurance, has predominated in medical care (over 50 years of Medicare and Medicaid in the US; over 20 years of national health insurance in Israel) while private insurance and self-pay have predominated in dental care. Public insurance is predicated on a liberal or social democratic political philosophy, while private insurance and individual responsibility for care are, in sharp contrast, predicated on conservative and libertarian philosophies.

While most of U.S. oral health care is privately financed through either employer-based coverage or through self-pay, U.S. children of poor and working-poor families are the exception to the rule. They are publicly insured through Medicaid and the Children’s Health Insurance Program (CHIP), both of which currently mandate comprehensive dental services that are tax-funded [4] and delivered predominantly in private offices. In Israel, the longstanding exclusion of dental care from the government-financed health system (which we acknowledge is increasingly complemented by a robust private-pay system) had raised questions of equitable access to dental care for children. This was the condition that led the “policy entrepreneur” to push pediatric dentistry into the managed care medical structures created by the Israeli National Health Insurance Law.

Indeed, this push, which “challenged the equilibrium” in Israeli dentistry, was opposed by “an influential group,” the Israeli Dental Association, and the policy entrepreneur prevailed by leveraging “civil organizations and researchers” as well as his own official authority. Analogously, organized dentistry in the U.S. has actively opposed expansion of governmental engagement. This has been evidenced in its active opposition to inclusion of dental coverage in Medicare in the 1960s, reluctance to promote a dental mandate for children in CHIP in the 1990s, support for “free-standing” dental insurance separate from medical insurance in the Affordable Care Act in the 2000s, and its current opposition to dental therapists detailed below [5–7]. Yet, as in the Israeli case, the “balance of power” has shifted over time; policy entrepreneurs like the Children’s Dental Health Project, Oral Health America, the Santa Fe Group, foundations, activist dentists, advocates for the poor, and individual legislators have overwhelmed traditional self-interests. These organizations and others - at different times and with different motives - have variously secured a dental mandate in CHIP, advanced medical-dental coverage integration, reformed state dental practice acts to expand scope of practice for allied dental personnel, and are now creating momentum for dental therapists and a dental benefit in Medicare.

## Philanthropic foundations as policy entrepreneurs

In the US, philanthropic foundations have acted as, or have supported, health policy entrepreneurs since at least 1927, when eight foundations provided funding to cover research and administrative costs for the Committee on the Costs of Medical Care. This funding enabled the committee’s 48 self-nominated individuals interested in reform, including physicians, public health officials, hospital administrators, dentists, economists, and others, to maintain independence from any established stakeholders. By 1932, 23 major reports had been issued by the Committee [8]. Within its analysis of the entire health system, they presented a vision of dental care as part of comprehensive health services. Among its recommendations were that (1) health care services should be largely provided by organized groups of physicians, dentists, nurses, pharmacists, etc., and (2) the cost of health care should be addressed on a group-payment basis, using both insurance and taxation.

However, these recommendations were delivered to a society unprepared to reorganize health care using an economic model rather than the autonomous, cottage industry model supported by the medical and dental professions. With regard to dental care today, it has only been over the past few decades that this vision, has come to pass (and still, mostly only for children) through the influence of various agents challenging the status quo.

Foundations continue to play a critical role in the advancement of a modest policy proposal that has recently brought to the U.S. the dental therapist, a type of allied dental professional trained to provide a narrow set of commonly-needed procedures. Dental therapists have provided dental services in 54 other countries, starting in New Zealand in 1921 [9]. Initially focused on school children, some countries have expanded their role to include care for adults in the private sector. Dental therapists work under the general supervision of dentists and are considered in the U.S. to be mid-level providers, similar to physician assistants in medicine. They provide preventive and routine restorative care, such as filling cavities, placing temporary crowns, and extracting loose teeth. Where permitted, dentists hire and supervise dental therapists to expand care to more patients, grow their practices, and provide treatment to underserved, at-risk populations in settings that are convenient for patients, such as schools or nursing homes [10].

In spite of the existence of national accreditation standards for the education and training of health care professionals, regulations defining supervision levels and scopes of practice in the U.S. are state-determined and vary widely from state to state. Most Americans depend on policy at the state level to influence the availability of care. Over time, many states have altered their scope-of-practice and supervision regulations to allow a broader range of competent oral health care professionals to perform a wider range of procedures under various levels of supervision.

American Indian and Alaska Native tribal sovereignty makes such action exempt from state laws in areas under their jurisdiction. Sovereignty enabled the Alaska Native Tribal Health Consortium to introduce dental therapists to provide care to Alaska Natives in tribal villages in 2005 over the objections of the Alaska Dental Association [11]. Inspired by the success of this program, several foundations convened researchers, public relations and marketing firms, and political strategists to build support that could be deployed to assist champions of dental therapy at the state level [7].

In each state where dental therapist policy was considered, it has been vehemently opposed by organized dentistry that objects to non-dentists performing irreversible surgical procedures without direct supervision by dentists [12]. The American Dental Association has provided considerable financial support and political expertise to state dental societies to undermine proponents.

The roadmap for policy entrepreneurship by foundations was provided by a 2011 report of the Institute of Medicine (now the National Academy of Medicine). It recommended that state dental practice acts should allow allied dental professionals (1) to practice to the full extent of their education and training, (2) to work in a variety of settings under evidence-based supervision levels, and (3) to collaborate with supervising dentists through remote technology [13]. Lacking any authority in its own right, the Institute proposed that foundations, professional organizations, and public policy organizations conduct research on the practice acts and their impact on access to dental services and issue “best practices” briefs to highlight state actions and their impacts on equitable access. Given the political independence of foundations afforded by their financial resources, they have been able to recruit and support activities by individuals and organizations, who are generally much less politically powerful than organized dentistry, to invest their time, energy, and reputations to function as policy entrepreneurs.

The principal policy entrepreneurs for the promotion of dental therapists have been supported by the W. K. Kellogg Foundation and The Pew Charitable Trusts, foundations with broad national and international agendas. They have partnered with state-based foundations in the states where windows of opportunity were identified by local policy entrepreneurs. Recognizing the inefficiency of having each state develop its own standards for training dental therapists, foundations supported the achievement of two critical milestones. First, the W. K. Kellogg Foundation and the Josiah Macy Jr. Foundation provided grants to the American Association of Public Health Dentistry, which convened an 11-person academic panel that was selected for expertise, experience, and in-depth knowledge of dental education. It produced a series of papers that highlighted proposed curriculum guidelines for the training of dental therapists, not as independent practitioners, but as members of the dental team, to help meet growing U.S. oral health needs, particularly among underserved populations [7].

Second, advocates petitioned the Commission on Dental Accreditation, the accrediting body for academic dental programs, to affirm educational standards for dental therapy training, and a process for accreditation, which was finalized in August, 2015 [14]. The establishment of accreditation standards promotes consistent levels of training across institutions, assures competency among dental therapists nationwide, removes the burden on states to develop their own standards for training, and provides the legitimacy necessary to encourage academic institutions to launch training programs.

Policy entrepreneurs have also recruited two other influential forces to disrupt the network equilibrium and challenge working assumptions. While it has no authority to regulate state legislatures, the Federal Trade Commission has echoed Mill’s Harm Principle by questioning whether overly restrictive regulations—that protect dentists’ financial interests—preclude the countervailing benefit to society— increased access to care [15]. Think tanks that promote free-market solutions to social and economic problems, such as the Heartland Institute, and advocacy organizations, such as Americans for Prosperity-Kansas, argue that therapists can increase the output of basic dental services, enhance competition, reduce costs, and expand access. A poll conducted by Americans for Tax Reform, which framed the opportunity in those terms, revealed strong support for mid-level dental providers across all key demographic groups independent of political party affiliation [16].

In 2009, the state of Minnesota authorized the training and practice of dental therapists to care for underserved segments of its population. The state’s first dental therapists entered practice in 2011. Two subsequent state government reports indicate that the dental therapy workforce is growing, practicing safely and working mostly in private dental offices, and apparently fulfilling statutory intent by serving predominantly low-income, uninsured and underserved patients [17, 18]. Maine enacted legislation in 2014 authorizing dentists to hire dental therapists, and that state is now working to implement the law and create a training program. In June, 2016, Vermont became the third state to allow dentists to hire these midlevel providers [19]. American Indian tribes in Oregon and Washington have also secured changes in state law allowing them to hire dental therapists [20, 21].

While both the American workforce reform and the Israeli insurance reform illustrate how policy entrepreneurs can challenge the equilibrium of a policy network, the role of foundations in the U.S. contrasts with the example illustrated by Cohen in several ways. Much of the Israeli entrepreneur’s accomplishment was attributable to his influence as a government cabinet member. It is clear that his influenced waned when general elections put him out of government leadership, even though he continued to serve as a member of the Knesset. In the U.S. example, success cannot be attributed to any single individual or entity, as policy had to be made in multiple jurisdictions in response to several windows of opportunity. Critical to the foundations’ disruptive success was their capacity to match organized dentistry’s financial resources and to use those resources to develop objective evidence that refutes organized dentistry’s claims, to enlist political strategists in developing persuasive arguments for policymakers, and to engage public relations firms to build public support for dental therapists.

## Conclusion – The Future

Perhaps the traction gained by opponents to organized dentistry’s “protracted stagnation” lies not as much in their expanding organization, voice, and policymaking acumen as in shifting values in their societies. In the U.S., high rates of personal healthcare-associated bankruptcy coupled with dissatisfaction with the cost and quality of care is increasing societal appreciation that health systems need to deliver better health outcomes at lower costs. The public and their policymakers now aspire to care that is more accountable, accessible, patient- and family-centered, and equitable. The idea of “value based purchasing” which requires assessment of quality and outcomes, is gaining traction not only among policymakers who fear the economic consequences of an ever-expanding healthcare tab but among the press that informs and reflects the public. This would constitute a shift in emphasis from volume to value and from measuring inputs to measuring outputs.

Changes made to pediatric dental care in the Israel parallel changes underway in the U.S. to systematize a disparate, disaggregated delivery system. Government payments provide leverage pressuring at least some dental care to be provided through HMO-like networked structures. Further changes may occur when payers seek value-based payment approaches that hold providers accountable for health outcomes. The most visible driver for this change in the U.S. has been the Patient Protection and Affordable Care Act (aka, ACA or “Obamacare”), which codified and accelerated, rather than created, changes in U.S. healthcare financing and delivery. Since these health system changes are well underway regardless of the ACA, U.S. health reform—with its shift from volume to value and its response to public demands for accountability, quality, accessibility, and equity—will continue albeit perhaps at a slower pace even as the law is modified or replaced during the Trump Administration. Yet as in Israel, with the exception of pediatric dental care, the dental profession is largely exempted from these changes. So what exactly is happening and where might it take pediatric dental care in the future?

As public and private payers demand value from healthcare providers for their dollars or shekels, they will increasingly utilize performance metrics to judge that value [22]. For pediatric dentistry, such metrics may include objective assessments of children’s oral health status and outcomes of care rather than catalogues of procedures provided; parental and older children’s reports of satisfaction with care; parental and older children’s knowledge of oral disease prevention; and self-reports from parents and older children of oral health quality of life, oral health status, and oral health behaviors. In short, payment can be expected to become increasing matched with ‘upstream’ measures of oral health determinants and downstream measures of oral health status, rather than to dental treatment procedures.

When modern dental teams are financially rewarded for pediatric oral health outcomes rather than procedures provided, they can be expected to triage groups of children by disease risk and intervene selectively using approaches like the American Academy of Pediatric Dentistry’s ‘care paths’; turn their attention to behavioral, social, and environmental oral health determinants; develop meaningful and effective family-level educational endeavors; engage social workers, health educators, dieticians, and peer counselors to facilitate daily healthful behaviors; and integrate their services with primary medical care [23]. When rewarded for improving oral health, dental teams that care for children can be expected to seek out high-risk children for whom they can demonstrate oral health gains rather than prioritize low-risk children for whom intensive use of scarce resources provides relatively little value when measured as oral health improvements. Behavioral and pharmacologic approaches to caries management will predominate over restorative approaches and wasteful allocation of resources (e.g., semiannual prophylaxis and topical fluoride treatments for low-risk children) will be reduced. The first step in promoting these long-endorsed, but little-utilized, approaches is a change in payment incentives which, in turn, depend on the kinds of policy development now underway in both the U.S. and Israel.

## Abbreviations

CHIP: Children’s Health Insurance Program
HMO: Health Maintenance Organization
IJHPR: Israeli Journal of Health Policy Research
US: United States of America
